# Decitabine plus CLAG chemotherapy as a bridge to haploidentical transplantation in the setting of acute myeloid leukemia relapse after HLA-matched sibling transplantation: a case report

**DOI:** 10.1186/s12885-019-5464-0

**Published:** 2019-03-18

**Authors:** Mengqi Jin, Yongxian Hu, Wenjun Wu, Yi Luo, Yamin Tan, Jian Yu, Aiyun Jin, Luxin Yang, He Huang, Guoqing Wei

**Affiliations:** Bone Marrow Transplantation Center, The First Affiliated Hospital, School of Medicine, Zhejiang University, Hangzhou, People’s Republic of China

**Keywords:** D-CLAG, Relapse, Acute myeloid leukemia, Bridge chemotherapy, Second transplantation

## Abstract

**Background:**

Patients with relapsed/refractory acute myeloid leukemia after hematopoietic stem cell transplantation (HSCT) have a poor prognosis, with a 2-year survival rate of 14%. The optimal treatment for these patients remains unclear. To treat these patients, we designed a new salvage regimen consisting of decitabine, cladribine, cytarabine, and granulocyte-stimulating factor (D-CLAG).

**Case presentation:**

Here, we describe a case of acute monocytic leukemia with a complex karyotype in a 38-year-old female patient who relapsed after her first HSCT, which was performed using a matched sibling donor. The patient did not respond to standard induction chemotherapy and subsequently achieved complete remission with the D-CLAG regimen. No severe hematological or extramedullary toxicity was observed. Subsequently, the patient received a second D-CLAG regimen as a bridge therapy and directly underwent haploidentical related HSCT. Following HSCT, the marrow showed complete hematologic and cytogenetic remission. Currently, 1 year after transplantation, the patient’s general condition remains good.

**Conclusions:**

This case suggests that the D-CLAG regimen can be an option for reinduction in relapsed refractory AML patients as a bridge to transplantation. Nevertheless, further research will be required in the future as this report describes only a single case.

## Background

Based on previous studies, 30–37% of patients with acute myeloid leukemia (AML) relapse after transplantation within 5 years [1, 2]. Of the AML patients who relapse after transplantation, only 10–32% achieve new remission [2, 3]. Therefore, these patients face a very poor prognosis with a 2-year survival rate of 14% [2, 4]. The optimal treatment for relapse of acute leukemia after hematopoietic stem cell transplantation (HSCT) remains unclear. Usually, the treatment options for these patients are limited.

The cladribine, cytarabine, and granulocyte-stimulating factor (CLAG) regimen has been used for the treatment of relapsed/refractory AML either alone or followed by HSCT, resulting in a complete remission (CR) rate of 49–62% [5, 6]. The key chemotherapy drug in the CLAG regimen is cladribine, which is an adenosine deaminase-resistant analog of adenosine that induces apoptosis in myeloid cells primarily by interfering with DNA synthesis [7]. In addition, cladribine may modulate the bioactivation of cytarabine. Interestingly, mononuclear leukemia cells appear to be more sensitive than other leukemia cells to deoxyadenosine analogs because these analogs induce the differentiation of myelomonocytic leukemia cells [8]. However, the CR rate declines in patients who relapse after HSCT [4]. Therefore, adjusting the CLAG regimen is urgent for obtaining a higher CR rate and improving efficacy. Here, we combined another chemotherapy with CLAG to strengthen its antileukemia activity in an AML patient who relapsed after the first HSCT.

Increasing evidence emphasizes the importance of epigenetic modifications in the pathogenesis of acute leukemia. In contrast to DNA mutations, epigenetic changes, such as methylation or acetylation, can be reversed pharmacologically [9]. The purine analog decitabine acts primarily by inhibiting DNA methyltransferase and improving epigenetic deterioration. Furthermore, decitabine can sensitize AML cells to conventional chemotherapeutics, such as cytarabine and daunorubicin [10]. Several studies have found that decitabine is especially beneficial in AML patients with complex karyotypes [11]. Therefore, some researchers have indicated that decitabine is a well-tolerated treatment for patients with relapsed/refractory AML, even in cases with increased age and merged burden.

Although consensus regarding the optimal donor for a second transplantation is lacking, a previous study performed at our center indicated that the graft-versus-leukemia effect in high-risk leukemia patients is superior when haploidentical related donors are used compared with that when matched sibling donors or unrelated matched donors are used [12]. Based on the above information, we designed a salvage regimen for an AML-M5 patient who relapsed after her first transplantation. Decitabine followed by CLAG was used as the bridge chemotherapy. After CR, the same chemotherapy was used again prior to haploidentical HSCT. We attempted to perform the transplantation under a low tumor load and achieved success.

### Case presentation

A 38-year-old Chinese female was first admitted to our hospital in December 2011 due to a complaint of constipation for 1 month. Her diet and lifestyle were normal. She had no history of serious illness or family genetic diseases. During the physical examination, no abnormalities were identified. The peripheral blood counts revealed a white cell count of 1.3 × 10^9^/L, a hemoglobin level of 93 g/L, and a platelet count of 94 × 10^9^/L. The blood chemistry findings showed normal lactate dehydrogenase, C-reactive protein, and albumin levels. Her bone marrow was hypercellular, exhibited infiltration and included 91.5% blast cells comprising primitive monocytes and naive monocytes. The immunophenotype analysis showed that 54% of the cells were abnormal, and positive labeling for CD34, CD10, and CD71 and negative labeling for CD19 were observed. The overall findings were consistent with acute monocytic leukemia. G-banding revealed 45, XX, − 2, der(11)(p15) [3]/46,XX[16]/92,XXXX [1]. The genetic tests, including screens for FLT3, IDH1/2 and tp53 mutants, were all negative. The patient was diagnosed with high-risk acute monocytic leukemia. The patient did not respond to idarubicin and cytarabine (IA) or subsequent aclacinomycin, cytarabine, and etoposide (AAE). Then, the patient achieved CR following one additional AAE regimen as previously described. Furthermore, she received aclacinomycin and cytarabine (AA) twice, mitoxantrone and cytarabine (MA) once, and intermediate-dose cytarabine once as consolidation chemotherapy. Immediately thereafter, the patient underwent sibling HSCT from her HLA-identical sister in October 2012. The patient achieved continued CR but still exhibited microresidual disease of 0.01–0.05% in the following 4 years. Additionally, she did not suffer from acute or chronic graft-versus-host diseases after her first transplantation.

**Fig. 1:**
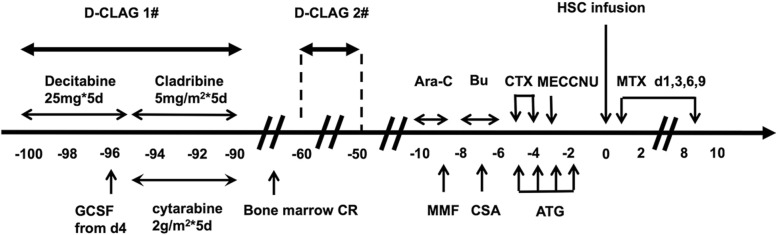
Flowchart of the two D-CLAG chemotherapies and haploidentical transplantation. Legends: D-CLAG: regimen consisting of decitabine, cladribine, cytarabine, and granulocyte-stimulating factor, Ara-C: cytarabine, Bu: busulfan, CTX: cyclophosphamide, MECCNU: methyl-N-(2-chloroethyl)-N-cyclohexyl-N-nitrosourea, HSC: hematopoietic stem cell, MTX: methotrexate, GSF: granulocyte-stimulating factor, CR: complete remission, MMF: mycophenolate mofetil, CSA: cyclosporin A, ATG: anti-thymocyte globulin

**Fig. 2:**
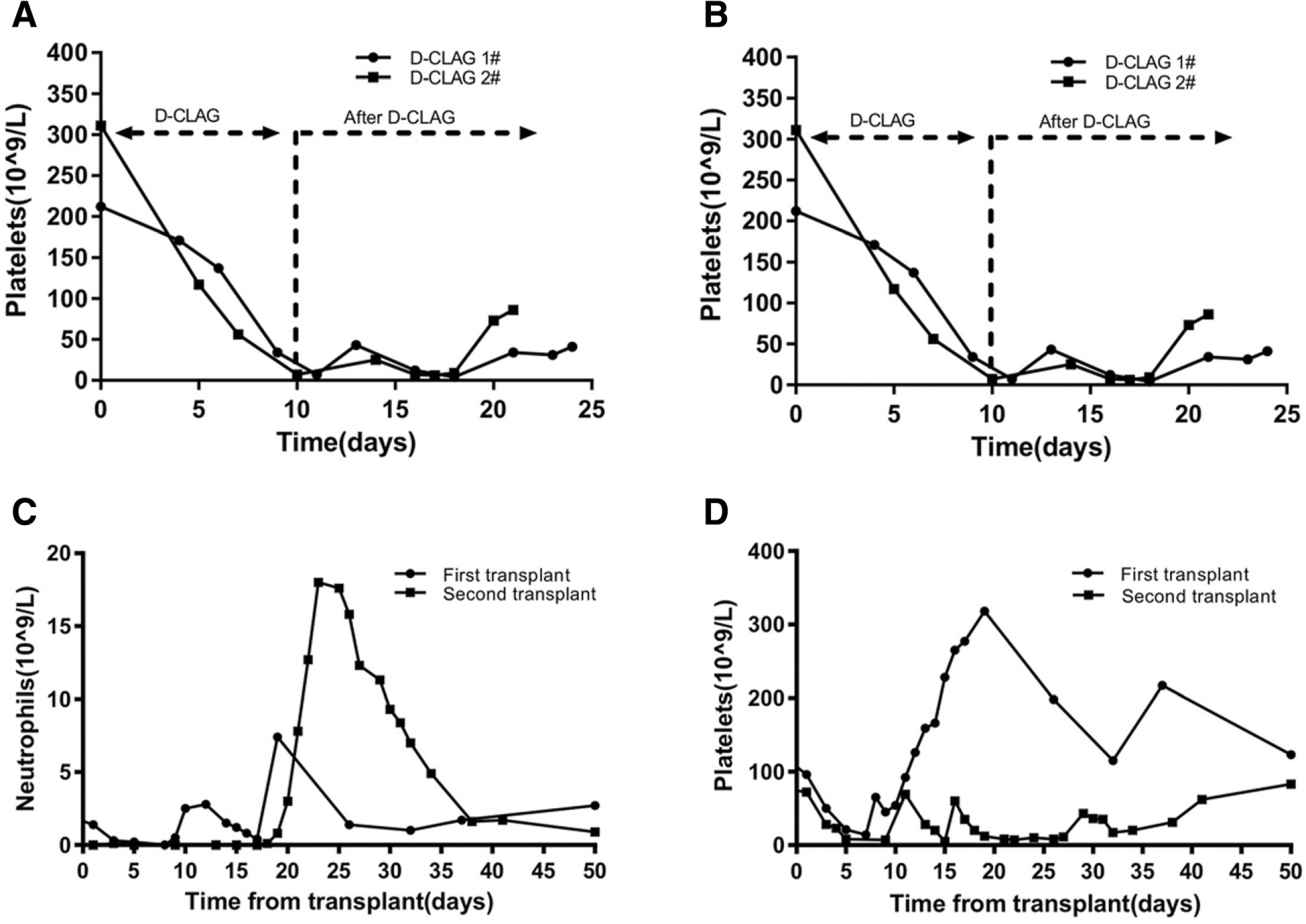
Recovery of neutrophils and platelets after chemotherapies and transplantations. Legends: **a** The recovery times required to achieve a normal neutrophil count after two D-CLAG regimens. **b** The recovery times required to achieve a normal platelet count after two D-CLAG regimens. **c** The recovery times required to achieve normal neutrophil levels after two transplantations. **d** The recovery times required to achieve normal platelet levels after two transplantations

The patient was admitted to our hospital again with complaints of fever and cough in April 2017. The peripheral blood counts revealed a white cell count of 4.8 × 10^9^/L, a hemoglobin level of 117 g/L, a platelet count of 170 × 10^9^/L and 10% abnormal cells. Her bone marrow was hypercellular, exhibited infiltration and included 51% blast cells comprising primitive monocytes and naive monocytes. The level of donor chimerism in her bone marrow was 47.9%. The genetic tests, including screens for FLT3, IDH1/2 and tp53 mutations, were all negative. Subsequently, the patient did not respond to mitoxantrone, cytarabine and etoposide (MAE) or donor lymphocyte infusion. Because the patient was refractory and exhibited AML relapse, subsequent D-CLAG chemotherapy (decitabine, 25 mg d1–5; cladribine, 5 mg/m^2^ d6–10; cytarabine, 2 g/m^2^ d6–10; and granulocyte-stimulating factor, 150 μg twice daily from d4 until the neutrophils exceeded 0.5*10^9^/L) was administered (Fig. 1). A bone marrow test performed 3 weeks after the D-CLAG regimen showed CR. Moreover, the neutrophils and platelets recovered quickly (Fig. 2a, b). Additionally, the patient did not suffer from any severe complications after chemotherapy. Subsequently, the patient was given one more D-CLAG and donor lymphocyte infusion. A bone marrow test performed 1 month after the second D-CLAG showed CR. During her follow up, she underwent haploidentical HSCT from her daughter in August 2017. The conditioning regimen consisted of cytarabine, busulfan, cyclophosphamide, methyl-N-(2-chloroethyl)-N-cyclohexyl-N-nitrosourea, and anti-thymocyte globulin. Methotrexate, cyclosporin A and mycophenolate mofetil were used for graft-versus-host disease prophylaxis. The numbers of mononuclear cells and CD34+ cells were 15.07 × 10^**8**^/kg and 5.68 × 10^**6**^/kg, respectively. The neutrophil and platelet engraftments were achieved on day 17 and day 29, respectively (Fig. 2c, d). A short tandem repeat analysis showed complete donor-type engraftment. She suffered gastrointestinal bleeding on day 20 but quickly recovered with supportive treatment. Epstein-Barr virus infection was observed 2 months after transplantation with a maximum Epstein-Barr virus DNA load of 3 × 10^**5**^ copies/ml. The patient did not develop a posttransplantation lymphoproliferative disorder, and the Epstein-Barr virus DNA load decreased below the normal level with intravenous rituximab. Following haploidentical HSCT, her marrow showed continuous CR, and the microresidual disease remained below 0.01%. Currently, one year after transplantation, the patient continues to be in good general condition.

## Conclusions

In this case, we chose D-CLAG as a bridge chemotherapy before haploidentical transplantation for a relapsed AML-M5 patient, representing a treatment regimen that has not yet been reported. Performing high-intensity chemotherapy before allogeneic transplantation eliminates residual tumor cells and inhibits the recipient’s immune system to facilitate the implantation of hematopoietic stem cells. This outcome confirms the efficacy of this regimen, and the duration of agranulocytosis and infection was acceptable.

In our case, the efficacy of D-CLAG was compelling. Previously, CLAG alone has been used for the treatment of relapsed/refractory leukemia at some research centers. The Polish Adult Leukemia Group investigated the activity of a combination consisting of cladribine (5 mg/m^2^), cytarabine (2 g/m^2^) and granulocyte-stimulating factor in 58 patients with resistant or relapsed refractory AML [13]. The rate of CR was 50%, and the overall survival at 1 year was 42%. Compared with chemotherapy in which CLAG is used alone, our regimen exhibited significant antileukemia activity in refractory AML. In our case, we added decitabine, which is a subtype of epigenetic modifiers, to the classic regimen after considering the complexity of the patient’s chromosomes. In addition to decitabine, other available epigenetic modifiers, such as IDH1 inhibitors, IDH2 inhibitors and DOT1L inhibitors, have been researched in suggested patient populations according to the identified mutations [14]. Furthermore, several novel drugs, including cytotoxic chemotherapy, protein kinase inhibitor antibody-drug conjugates, and cell cycle inhibitors, have been developed as replacements for conventional regimens [14, 15]. These emerging drugs have the potential to be combined with classic regimens, especially in patients with chromosomal or genetic abnormalities. Our chemotherapy approach can be regarded as a new option.

Regarding side effects, we found that the toxicity of the chemotherapy regimen applied in our case was very low. According to previous studies, the main toxicities of decitabine and CLAG are hematological, and extramedullary adverse reactions are very rare. Following our two D-CLAG chemotherapy regimens, the patient showed granulocyte deficiency at 8 and 12 days after chemotherapy. Additionally, the platelets returned to a standard level (> 20*10^9^/L) at 10 and 11 days after chemotherapy. Furthermore, the patient did not suffer from any infection at the granulocyte stage during either D-CLAG chemotherapy regimen. Following D-CLAG combined with haploidentical transplantation, the successful engraftment of granulocytes and platelets was achieved. The complications, including gastrointestinal hemorrhage, cytomegalovirus and Epstein-Barr virus infection, were temporary and quickly resolved within 7 days with supportive treatment. Moreover, no obvious graft-versus-host disease was observed during the one-year follow-up period. However, our article is limited because it is a single-case report, and we aim to apply the D-CLAG regimen to more relapse/refractory AML patients to fully evaluate its efficacy.

In summary, our results confirm the high antileukemic efficacy of D-CLAG as a bridge to haploidentical transplantation and that this regimen had acceptable toxicity. This case suggests that the D-CLAG regimen could be an option for reinduction in relapsed refractory AML patients as a bridge to transplantation. Nevertheless, because this is a single case report, further research will be required to validate the efficacy of this new regimen.

## Abbreviations

AML: acute myeloid leukemia
CD: cluster of differentiation
CLAG: cladribine, cytarabine, and granulocyte-stimulating factor
CR: complete remission
D-CLAG: decitabine, 25 mg d1–5; cladribine, 5 mg/m2 d6–10; cytarabine, 2 g/m2 d6–10; and granulocyte-stimulating factor, adjusted according to the situation
DNA: deoxyribonucleic acid
HLA: human leukocyte antigen
HSCT: hematopoietic stem cell transplantation
