# Correction to: Decitabine plus CLAG chemotherapy as a bridge to haploidentical transplantation in the setting of acute myeloid leukemia relapse after HLA-matched sibling transplantation: a case report

**DOI:** 10.1186/s12885-019-5626-0

**Published:** 2019-05-23

**Authors:** Mengqi Jin, Yongxian Hu, Wenjun Wu, Yi Luo, Yamin Tan, Jian Yu, Aiyun Jin, Luxin Yang, He Huang, Guoqing Wei

**Affiliations:** Bone Marrow Transplantation Center, The First Affiliated Hospital, School of Medicine, Zhejiang University, Hangzhou, People’s Republic of China

**Correction to: BMC Cancer**

**https://doi.org/10.1186/s12885-019-5464-0**

Following publication of the original article [1], the authors reported that the incorrect Fig. 2a was published in the article. The recovery times required to achieve a normal neutrophil count was omitted. The corrected Fig. 2 is given below.

**Fig. 2:**
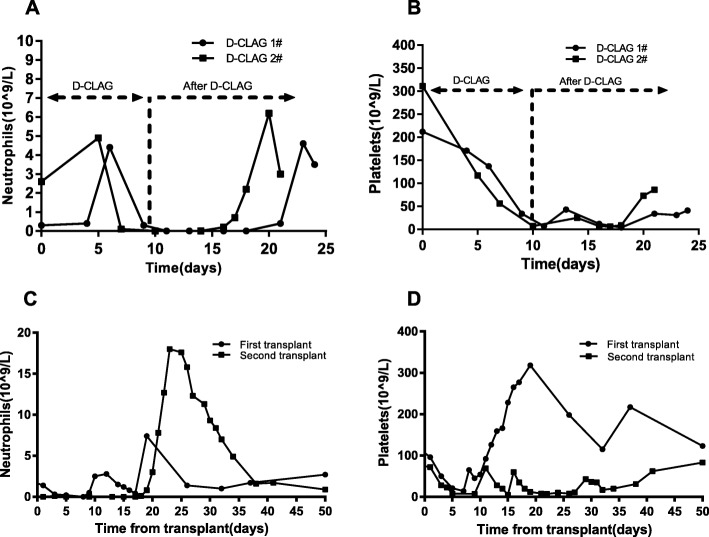
Recovery of neutrophils and platelets after chemotherapies and transplantations. Legends: **a** The recovery times required to achieve a normal neutrophil count after two D-CLAG regimens. **b** The recovery times required to achieve a normal platelet count after two D-CLAG regimens. **c** The recovery times required to achieve normal neutrophil levels after two transplantations. **d** The recovery times required to achieve normal platelet levels after two transplantations

Second, a formatting error was found in the section “Case presentation”. In the sentence “G-banding revealed 45, XX, − 2, der(11)(p15) [3]/46,XX[16]/92,XXXX [1]”, “[3, 16, 1]” were in fact part of complex chromosome abnormalities. However, [1] and [3] were regarded as parts of reference citations in the published article.
